# Pain in Hospital: A Real‐Word Data Analysis

**DOI:** 10.1002/nop2.70126

**Published:** 2025-02-28

**Authors:** Virginie Claivaz, Malik Benmachiche, Zeina Santoro, Fabienne Hadorn, Cédric Mabire

**Affiliations:** ^1^ Clinical Nurse Specialist, Valais Hospital Sion Switzerland; ^2^ Internal Medicine Service Lausanne University Hospital Lausanne Switzerland; ^3^ Lausanne University Hospital Lausanne Switzerland; ^4^ Institute of Higher Education and Research in Healthcare (IUFRS) Lausanne University Hospital and University of Lausanne Lausanne Switzerland

**Keywords:** hospital medicine, pain, pain assessment, pain management, prevalence

## Abstract

**Aim:**

The study aim was to describe the prevalence of pain, its assessment, and its associated characteristics in a university hospital in the French‐speaking part of Switzerland.

**Background:**

Despite many advances in its management, pain is still a common and persistent symptom in hospitals. Nurses have a central role in its management. Pain assessment is essential for optimal management; it is nonetheless made insufficiently and randomly.

**Design:**

The design was a monocentric correlational descriptive study.

**Methods:**

This study was based on the secondary analysis of routine data from 22,987 computerised health records of the medical and surgical wards between 1 November 2017 and 31 March 2019.

**Results:**

The results showed that the prevalence of pain was high in medical and surgical wards. Almost one‐fifth of the participants suffered from severe pain at least once during their hospital stay. There was no association between the presence of pain and hospital ward, but the likelihood of having severe pain increased if the participant was hospitalised in a medical ward. Close personalised pain monitoring should be promoted to prevent the onset of severe pain.

No Patient or Public Contribution.

## 1 Introduction

Pain remains a common and persistent symptom in hospitals, with a large prevalence of 37% to 68% (Becerra‐Bolaños et al. 2023; Damico et al. 2018; Lorenzo Allegue et al. 2023; Neuwersch‐Sommeregger et al. 2024; Notaro et al. 2021; Peterson and Söderlund Schaller 2022; Saunders et al. 2022) and that of severe pain as high as 19%–22% (Notaro et al. 2021; Peterson and Söderlund Schaller 2022; Saunders et al. 2022). These findings suggest that, despite many advances in nursing, pharmacology, and medicine, pain management is still inadequate. Pain affects patients' physical and psychosocial functioning, including personal hygiene, mobilisation, mood, sleep and appetite, and increases the risk of developing chronic pain, cardiorespiratory, pulmonary, gastrointestinal or renal complications (Becerra‐Bolaños et al. 2023; Gan 2017; Jabusch et al. 2015; Meissner et al. 2018, 2017; Neuwersch‐Sommeregger et al. 2024; Peterson and Söderlund Schaller 2022; Zoëga, Sveinsdottir, et al. 2015). It is associated with delayed recovery time, higher mortality and increased health‐care costs (Gan 2017; Meissner et al. 2018). Because of their position, nurses have a key role to play in pain management: first, by identifying the presence and the characteristics of pain, and then by planning nonpharmacological and pharmacological interventions to prevent or to control pain, and, finally, by regularly reassessing pain to adjust the care plan (Coll and Jones 2020; Glarcher et al. 2021; Malones et al. 2021; Sonneborn et al. 2022).

## 2 Background

Assessment is essential, as it enables the healthcare professional to be aware of the presence and severity of pain (Malones et al. 2021; Saunders et al. 2022; Schug et al. 2020; Song et al. 2015; Zoëga, Ward, et al. 2015). Pain needs to be assessed regularly according to the established standards on the specific ward (Neuwersch‐Sommeregger et al. 2024; Schug et al. 2020), but because of a lack of evidence, no general or specific practice recommendations are available to determine the optimal frequency of assessment, regardless of ward. Although pain assessment is part of the daily routine in hospitals, performed at least once a day, systematic pain assessment without individualisation seems to have little impact. Two studies described a positive association between the assessment and documentation of pain intensity and the appropriateness of the treatment's administration (Lorenzo Allegue et al. 2023; Zoëga, Ward, et al. 2015). Otherwise, no association has been found between the pain intensity score, interventions and the number of assessments performed, suggesting that assessment is often done randomly, insufficiently, and without validated assessment tools; moreover, it is not always documented in the medical record (Coll and Jones 2020; Heikkilä et al. 2016; Lorenzo Allegue et al. 2023; Song et al. 2015).

Nonpharmacological or pharmacological pain interventions should be provided by taking an individualised and multimodal approach that depends on the outcome of the pain assessment. In practice, however, the prescription, administration and documentation of antalgic interventions are often lacking, even when pain has been documented (Heikkilä et al. 2016; Song et al. 2015; Zoëga, Ward, et al. 2015). Of the many intervention options, the administration of analgesic drugs remains the most frequently applied (Ayhan and Kurşun Kural 2020; Damico et al. 2018; Zoëga, Ward, et al. 2015) with nonpharmacological interventions being scarce and limited in variety, despite ample evidence of their effectiveness (Ayhan and Kurşun Kural 2020; Song et al. 2015; Zoëga, Ward, et al. 2015). Regardless of the literature recommendations, multimodal interventions are lacking (Damico et al. 2018; Zoëga, Ward, et al. 2015).

The availability of pain management guidelines notwithstanding, the prevalence of pain in hospitals remains high. The prevalence experienced by patients depends on their characteristics and the ward specialty. It appears to be higher in medical and surgical wards than in other hospital wards (Beck et al. 2019). Comparisons between studies are difficult because there is no consensus on how to measure the prevalence of pain in hospitals (Glarcher et al. 2021).

Pain management is an important quality issue. Quality improvement programs appear to play a central role in the adoption of evidence‐based practices, such as pain assessment (Glarcher et al. 2021; Heikkilä et al. 2016). Pain management standards can be identified by measuring structure, process, and outcome quality indicators and analysing them in daily practice (Glarcher et al. 2021; Song et al. 2015). At the moment, however, studies are based on small real‐world samples or include only those participants with their capacity for judgement. Although no consensus is available on the association between pain and medical specialty, an association may be found if all inpatients in a hospital are studied. Here, we analysed structured routine data to provide us with more information about the application of evidence‐based recommendations in practice. The aim of this study was to describe the prevalence of pain and its management, as well as the correlation between the pain intensity score and patient and hospitalisation characteristics in a university hospital in Switzerland.

## 3 Method

### 3.1 Design, Setting and Sample

This monocentric retrospective correlational descriptive study was conducted at the Lausanne University Hospital, a large teaching hospital in the French‐speaking part of Switzerland (1560 beds). The research team consisted of five members: two MScSI students whith over 5 years of nursing experience whithin the institution, a senior physician, a nurse responsible for clinical improvement program, and a professor from the Faculty of Nursing IUFRS who supervised the research. We performed a secondary analysis of structured routine data from the hospital's computerised data warehouse, called Soarian. The data analysed pertained to the documentation of pain assessment and management recorded by bedside nurses. Medical and administrative records from medical and surgical departments were analysed. No sampling method was used. Inclusion criteria included being an adult inpatient, being hospitalised for more than 48 consecutive hours between 1 November 2017, and 31 May 2019, and providing consent to the further use of the patient's clinical data.

### 3.2 Data

Data were extracted by a dedicated team using the inclusion criteria. The data obtained were concatenated and aggregated to obtain an analysis table. Someone who was not involved in the study ensured that participants who refused to sign the general consent form or who had a document stating that they refused to participate in a research project were excluded. Since January 2013, all patients hospitalised in the CHUV are asked for general consent that allows future use of medical records and blood tests performed during their hospitalisation. For patients who did not give explicit consent, reuse of the data was authorised if there was no clear refusal, according to art 34 of the Swiss law on human research. Patients who denied reuse of their data were excluded. The final data obtained were then anonymised through serial coding of six randomly generated digits. Therefore, it was not possible to assess how many patients were excluded. In total, 22,987 file extracts were performed over the 19 months of data collection and were provided to the investigators.

### 3.3 Outcomes and Measures

The variables corresponded to the pain score of patients with pain or severe pain. Missing data were identified by a non‐zero value to ensure the integrity of results and to quantify the number of assessments conducted nonetheless. The primary outcomes were pain and severe pain prevalence. The secondary outcome was the difference in pain and severe pain prevalence between medical and surgical wards, including patient characteristics.

The selected patient characteristics were age, gender, number of active diagnoses and the presence of the following comorbidities: any tumours, any digestive diseases, or any respiratory diseases. The selected hospitalisation characteristics were type of ward (categorised as medical or surgical), hospital length of stay (in days), ward length of stay (in days), presence of documented pain, type and number of pain assessments, nonpharmacological and pharmacological interventions and number of surgical interventions. Specialties corresponding to medical or surgical wards are listed in the Appendix. Internal medicine corresponded to 25.92% of ward stays analysed, visceral surgery to 17.38% and otolaryngology to 9.34%. A patient could move between different wards during their hospital stay. To be counted as a ward stay, the patient must have spent at least 8 h on the ward.

In this hospital, nine different tools were used to assess and document pain intensity in the patients' record: four tools for self‐assessment scales (visual analog scale, numeric rating scale, verbal rating scale, face pain rating scale (Ferreira‐Valente et al. 2011; Gregory 2019; Karcioglu et al. 2018) and five tools for hetero‐assessment Critical‐Care Pain Observation Tool (Gélinas et al. 2006); Algoplus (Michel 2009; Rat et al. 2011; Wary and Villard 2006); Doloplus (Michel 2009); Behavioural Assessment of Pain in the Elderly (Michel 2009; Wary and Villard 2006); Face, Legs, Activity, Cry, Consolability—while initially developed for paediatric use, this scale has been validated for adult patients with communication difficulties, particularly those with cognitive impairments (Voepel‐Lewis et al. 2010). In this hospital, cut‐offs for the pain intensity self‐rated scales were set at a score greater than or equal to 3 out of 10. A score of greater than or equal to 3 required the establishment of an antalgic intervention. Severe pain corresponded to a score of greater than or equal to 7 out of 10. In these extractions, only 0.26% of the tools used to assess pain intensity were documented with a hetero‐evaluation scale. For this reason, only self‐assessment scores were retained in the following analysis.

### 3.4 Statistical Analysis

Descriptive statistics were used to describe demographic data and pain management variables. The prevalence of pain and severe pain were calculated for the whole sample and by the ward specialty (medical or surgical). In this study, the prevalence of pain was defined as the ratio between the number of ward stays with at least one documented pain score equal to or greater than 3 and the total number of ward stays. The prevalence of severe pain was defined as the ratio between the number of ward stays with at least one pain score equal to or greater than 7 and the total number of ward stays. Prevalences were compared by using a chi‐square test of independence. Univariable and multivariable linear regression and logistic regressions were performed. Prior to analysis, regression assumptions were tested. For linear regression, normality of residuals was assessed using skewness/kurtosis tests, homoscedasticity was verified using the Breusch–Pagan test, and multicollinearity was checked using the variance inflation factor (VIF). For logistic regression, model fit was evaluated using the Hosmer–Lemeshow test and multicollinearity was assessed using VIF. All confounding variables were included in these tests. The significance level was set at *p* < 0.05. Stata/MP 15.1 statistical software was used for analyses.

### 3.5 Ethical Consideration

This study and its procedure were approved by the ethics committee (Commission cantonale d'éthique pour la recherche sur l'être humain, CER‐VD) in August 2019 (N° 2019–00893), and no further specific consent was required from the patients, as only already available administrative data were used.

## 4 Results

### 4.1 Hospital and Ward Stays

A total of 22,987 inpatients were included, of which 31,968 ward stays were analysed, indicating that some patients had multiple hospital stays during the analysis period. The proportion of men was 55.60%, and the median age was 69.94 years. The median hospital length of stay was 7.78 days (Table 1).

**TABLE 1:** Demographic and clinical variables among surgical and medical wards (*N* = 31,968 ward stays).

Variable	Medical wards (*N* = 14,779)				Surgical wards (*N* = 17,189)				*p*
	Med	IQR	*n*	%	Med	IQR	*n*	%	
Age (years)	73.65	22.95			65	22.24			0.01^a^
Men			7601	51.43			10,594	61.63	0.01^b^
Number of active diagnoses	12	9			7	8			0.01^a^
Tumours			2166	14.87			5342	31.38	0.01^b^
Digestive diseases			1389	9.53			3647	21.43	0.01^b^
Respiratory diseases			2518	17.28			1090	6.40	0.01^b^
Ward stay with at least one surgical intervention			3870	20.95			14,601	79.05	0.01^b^
Ward length of stay (days)	6.13	9.59			3.03	4.22			0.01^a^

Abbreviations: Med, median; IQR, interquartile range; *n*, absolute frequency; %, relative frequency.

^a^ Wilcoxon test.

^b^ χ^2^.

### 4.2 Pain Assessment, Documentation and Prevalence

At least one pain assessment was documented for 96.35% of ward stays (Table 2). The median number of pain assessments documented per day was 2.36 (interquartile range = 1.80). Pain scales were used in 84.59% of the documented assessments. The most commonly used standardised pain scales were the verbal pain intensity scale (44.70%) and the numerical rating scale (44.46%). According to an adjusted linear regression, pain was assessed less frequently in medical wards than in surgical wards (*ß* = −6.79, 95% confidence interval [CI] [−7.01, −6.47]) (Table 3).

**TABLE 2:** Prevalence and pain characteristics under study (*N* = 31,968 ward stays).

Variable	Medical wards (*N* = 14,779)				Surgical wards (*N* = 17,189)				*p*
	Med	IQR	*n*	%	Med	IQR	*n*	%	
Pain prevalence (3–10)			7635	51.66			10,541	61.32	0.01^b^
Severe pain prevalence (7–10)			2901	19.63			3121	18.16	0.01^b^
At least one pain assessment			14,070	95.20			16,731	97.34	0.01^b^
Daily pain assessment	1.98	0.65			3.39	2.08			0.03^a^
At least one pharmacological intervention			8834	59.77			14,319	83.30	0.01^b^
At least one nonpharmacological intervention			1593	10.78			1308	7.61	> 0.01^b^
At least one pharmacological and one nonpharmacological intervention			1.293	8.75			1.236	7.19	> 0.01^b^

Abbreviations: Med, median; IQR, interquartile range; *n*, absolute frequency; %, relative frequency.

^a^ Wilcoxon test.

^b^ χ^2^.

**TABLE 3:** Linear regression of number of pain assessments (*N* = 31,968 ward stays).

Variable	Univariable regression		Multivariable regression	
	*β*	95% CI	*β*	95% CI
Ward length of stay (days)	1.98	[1.96, 2.00]	2.18	[2.16, 2.20]
Medical ward			−6.79	[−7.01, −6.47]
Number of surgical interventions			0.57	[0.50, 0.63]
Administration of analgesics			1.66	[1.37, 1.95]
Age (years)			−0.02	[−0,02, −0.01]
Men			−0.18	[−0,43, 0,07]
Number of active diagnoses			0.05	[−0,43, −0.07]
Tumours			2.89	[2.57, 3.20]
Digestive diseases			2.05	[1.69, 2.42]
Respiratory diseases			1.32	[0,91,1.73]

Abbreviations: CI, confidence interval; *β*, regression coefficient.

The prevalence of pain during the hospital ward stay was 56.86%; the prevalence of severe pain was 18.84%.

### 4.3 Pain Management

Analgesics were administered in 72.43% of ward stays, nonpharmacological interventions were provided in 9.07%, and both analgesic and nonpharmacological interventions were administered in 7.91%. The most commonly administered drugs were paracetamol (42.84%), morphine (13.35%) and metamizole sodium (10.67%).

### 4.4 Secondary Outcome: Multivariable Subgroup Analysis

According to an adjusted logistic regression, analgesics were given more often when pain was documented (odds ratio [OR] = 6.46, 95% CI [6.07, 6.88]) (Table 4). Men had less pain than women (OR = 0.73, 95% CI [0.69, 0.77]). Participants with a respiratory disease had less pain than those without respiratory disease (OR = 0.90, 95% CI [0.83, 0.98]). There was no difference in the probability of having pain between medical and surgical wards (OR medical versus surgical 1.01, 95% CI [0.94, 1.08]).

**TABLE 4:** Logistic regression of pain (*N* = 30,404 evaluations).

Variable	Univariable regression		Multivariable regression	
	OR	95% CI	OR	95% CI
Medical ward	0.67	[0.64, 0.70]	1.01	[0.94,1.08]
Ward length of stay (day)			1.00	[0.99, 1.08]
Administration of analgesics			6.46	[6.07, 6.88]
Number of pain assessments			1.05	[1.05, 1.06]
Number of surgical intervention			0.99	[0.98, 1.01]
Age (years)			0.98	[0.98, 0.98]
Men			0.73	[0.69, 0.77]
Number of active diagnoses			1.01	[1.00, 1.01]
Tumours			1.03	[0.96, 1.10]
Digestive diseases			1.00	[0.92, 1.08]
Respiratory diseases			0.90	[0.83, 0.98]

Abbreviations: CI, confidence interval; OR, odds ratio.

The risk of having severe pain was higher in a medical than in a surgical ward (OR = 1.46, 95% CI [1.35, 1.58]) (Table 5). Analgesics were more likely to be administered when severe pain was documented than when it was not (OR = 6.59, 95% CI [5.91, 7.36]). Men had less severe pain than women did (OR = 0.73, 95% CI [0.69, 0.78]). Participants with a respiratory disease had less severe pain than those without respiratory disease did (OR = 0.88, 95% CI [0.80, 0.98]).

**TABLE 5:** Logistic regression of severe pain (*N* = 30,765 evaluations).

Variable	Univariable regression		Multivariable regression	
	OR	95% CI	OR	95% CI
Medical ward	1.10	[1.04, 1.16]	1.46	[1.35, 1.58]
Ward length of stay (day)			1.00	[1.00, 1.01]
Administration of analgesics			6.59	[5.91, 7.36]
Number of pain assessments			1.02	[1.02, 1.02]
Number of surgical interventions			0.99	[0.98, 1.01]
Age (years)			0.98	[0.98, 0.98]
Men			0.73	[0.69, 0.78]
Number of active diagnoses			1.03	[1.03, 1.04]
Tumours			0.96	[0.89, 1.04]
Digestive diseases			0.94	[0.86, 1.03]
Respiratory diseases			0.88	[0.80, 0.98]

Abbreviations: CI, confidence interval; OR, odds ratio.

## 5 Discussion

The aim of this study was to describe the prevalence of pain and severe pain, the management of pain in hospitalised patients, and the correlation between the pain intensity score and patient and hospitalisation characteristics. The prevalence of pain was high in medical and surgical wards. Severe pain affected almost one‐fifth of the participants at least once during their hospital stay. There was no association between pain risk and ward, but the odds of severe pain increased if the participant was admitted to a medical ward. The odds of receiving antalgic interventions were higher if a pain assessment had been documented.

This study showed that the prevalence of severe pain was slightly higher in medical than in surgical wards, although the prevalence of pain was higher in surgical than in medical wards, a finding previously reported by Lorenzo Allegue et al. (2023). Several hypotheses could explain this finding.

Pain assessment being higher in surgical than in medical wards confirms previous findings (Lorenzo Allegue et al. 2023; Zoëga, Sveinsdottir, et al. 2015). Regular pain assessment allows nurses to be aware of the presence of pain and provide prompt treatment, thus preventing its likely worsening (Heikkilä et al. 2016; Zoëga, Sveinsdottir, et al. 2015). Patients in pain do not always think to notify the nurse, which is why assessment is a cornerstone of effective pain management (Lorenzo Allegue et al. 2023; Malones et al. 2021). Patients are more likely to share their pain when the nurse is available and consistently uses pain assessment tools, creating a sense of security (Malones et al. 2021). Adequate pain monitoring and its documentation ensure continuity of care, as healthcare professionals have sufficient information to intervene if necessary (Heikkilä et al. 2016). This difference in pain assessment could not be explained by the institution's quality improvement program, because it was the same in all wards of the institution where the study was conducted. However, it could be explained by nurses' adherence to evidence‐based practices. For nurses to adopt an evidence‐based practice, they need to see it as an added value. This added value could be demonstrated by the regular presence of pain specialists, such as the analgesia team, who frequently visited the surgical units. As specialists, they played a key role in ensuring adherence to evidence‐based practices and protocols by reminding staff of the importance of regular pain assessment through indirect coaching. This difference in pain assessment could also be explained by the fact that pain prevalence was a more important issue in the surgical wards than in the medical wards, which might also encourage surgical nurses to assess pain more frequently. Indeed, observing a patient in pain could prompt nurses to assess the patient's condition. The fact that surgical nurses assessed patients' pain more frequently could also be explained by surgical culture (Lorenzo Allegue et al. 2023; Zoëga, Ward, et al. 2015).

According to the literature, severe pain was more common on wards where patients were not treated according to pain management procedures (Beck et al. 2019; Damico et al. 2018). Guidelines were generally not available in medical wards, because the aetiology of pain is aspecific. In the hospital where the study was conducted, there were institutional recommendations for pain management in each ward, which provided a basis for supporting nurses' pain interventions. Guidelines, procedures, and specialist consultation services were resources that nurses could rely on when there was a gap in pain management (Zoëga, Ward, et al. 2015). However, this finding suggested that more resources were used on surgical wards, such as specific protocols, to raise awareness of pain among surgical nurses, encouraging its earlier assessment and, consequently, earlier pain management to prevent progression to severe pain.

Specific participant characteristics of the host specialty might also explain differences in the prevalence of severe pain. On surgical wards, the aetiology of pain, most often related to the surgical procedure itself, is better known than in medical wards, making the occurrence of pain more predictable and therefore probably easier to manage (Meissner et al. 2018). Patients with chronic pain, which is more difficult to manage, are usually hospitalised in medical wards (Beck et al. 2019). The number of active diagnoses is higher on medical wards than on surgical wards, which can lead to multiple causes of pain and complicate the choice of analgesics (Jabusch et al. 2015).

Finally, this difference in the prevalence of severe pain could also be explained by differences in the administration of analgesics between wards. Our study showed that analgesics were administered more frequently on surgical wards than on medical wards, confirming others findings (Becerra‐Bolaños et al. 2023; Lorenzo Allegue et al. 2023; Zoëga, Ward, et al. 2015). This difference in administration could be explained by closer monitoring of pain on surgical wards, as well as, as the literature suggested, by the fact that pain was part of daily postoperative care (Zoëga, Ward, et al. 2015). This difference could also impact patients' pain reporting: they are more likely to report their pain if nurses provide appropriate interventions to relieve it (Malones et al. 2021).

This study had two major strengths. It was the first study to examine the prevalence of pain and its associated characteristics in Switzerland across entire surgical and medical hospital wards of a hospital‐wide study. The heterogeneity of the sample was ensured by the size and the duration of data collection. The study methodology ensured that all ward stays were included.

This study also had several limitations. First, being based on retrospective data collected through electronic medical records, our findings might only partially reflect clinical reality. Gaps in documentation may not necessarily reflect actual practice, as some assessments might have been performed but not recorded, potentially leading to an underestimation of both pain prevalence and assessment frequency. Second, the institutional thresholds that led to analgesic interventions by nurses at the time of the study were not consistent with the literature recommendations and changed during data collection. Third, the assessment of pain intensity with only one pain dimension was not consistent with international recommendations. Finally, the results of this study cannot be generalised because of its monocentric nature.

### 5.1 Implications for Nursing Education, Practice and Research

Although improvements in pain management have been noted, nurses and the interprofessional team play a crucial role in pain management; however, their knowledge and adherence in pain assessment and management remain suboptimal. Regular pain assessment using validated tools is essential for accurately recognising and understanding patients' pain experiences enabling the creation of a personalised, preventive, and effective treatment plan to prevent adverse clinical outcomes. The results of this study suggest that coordinated and complementary actions should be developed to promote regular personalised pain assessments. To establish personalised assessments, it is important to restore meaning to this practice. Different strategies could be adopted: presentations with real data illustrating what has been done, what has not worked, and what could be done to improve pain management with institutional recommendations; coaching during daily practice; posters and pocket cards with friendly reminders of evidence‐based practices adapted by the clinicians to the local context. Special emphasis should be placed on medical wards where there are fewer specific recommendations.

For further research, it may also be interesting to investigate more dimensions of pain in order to have a broader understanding of personalised pain assessment and its management in hospitals.

## 6 Conclusion

In this study, three of five patients experienced pain at least once during their hospital stay; one of five experienced severe pain at least once during their hospital stay despite frequent pain assessments. The occurrence of pain and its management depend on patient characteristics, as well as on hospital characteristics and the extent to which the local context can be influenced. To improve pain management and the quality of care in hospitals, there is an urgent need to implement clinical projects to personalise assessment and care on the basis of evidence‐based practices.

## Ethics Statement

This study received ethical approval from the Cantonal Ethics Commission of the Canton of Vaud (Approval No. 2019–00893). As this research involves the utilisation of routine data, it was approved under Article 34 of the Swiss Federal Act on Research involving Human Beings. Explicit informed consent was obtained from all participants, ensuring their voluntary participation and understanding of the research procedures. The study was conducted in full compliance with the ethical principles and guidelines established by the Commission and adheres to the stipulations outlined in the Swiss Federal Act on Research involving Human Beings.

## Conflicts of Interest

The authors declare no conflicts of interest.

## Data Availability

The clinical data extracted and analysed during this study are not publicly available due to privacy and ethical constraints. However, the data are available from the authors upon reasonable request.

## Ward Stay Analyzed by Speciality

**Table:**

Specialty	Unit	*n*	%
Surgical	Visceral	5.556	17.38
	Otorhinolaryngology	2.985	9.34
	Thoracic	2.900	9.07
	Transplantation	936	2.93
	Urology	1.858	5.81
	Septic surgery	1.631	5.10
	Recovery room	367	1.15
	General surgery	956	2.99
Medical	Internal medicine	8.285	25.92
	Rehabilitation	2.309	7.22
	Intermediate care	2.229	6.97
	Geriatric	686	2.15
	Infectious illness	607	1.90
	Dermatology	442	1.38
	Palliative care	219	0.69
	Endocrinology and metabolism	1	0.00
	Nephrology	1	0.00
